# Bos d 13, A Novel Heat‐Stable Beef Allergen

**DOI:** 10.1002/mnfr.202200601

**Published:** 2023-06-30

**Authors:** Patricia Román‐Carrasco, Christoph Klug, Wolfgang Hemmer, Margarete Focke‐Tejkl, Marianne Raith, Isabella Grosinger, Peter Stoll, Santiago Quirce, Marta Sanchez‐Jareño, Mónica Martínez‐Blanco, Elena Molina, Veronika Somoza, Barbara Lieder, Zana Marin, Katharina Nöbauer, Karin Hummel, Ebrahim Razzazi‐Fazeli, Ines Swoboda

**Affiliations:** ^1^ Biotechnology Section FH Campus Wien Campus Vienna Biocenter University of Applied Sciences Vienna 1100 Austria; ^2^ FAZ‐Floridsdorf Allergy Center Vienna 1210 Austria; ^3^ Division of Immunopathology Department of Pathophysiology and Allergy Research Center for Pathophysiology Infectiology and Immunology Medical University of Vienna Vienna 1090 Austria; ^4^ Department of Allergy La Paz University Hospital, IdiPAZ Madrid 28046 Spain; ^5^ Instituto de Investigación en Ciencias de la Alimentación (CIAL, CSIC‐UAM) Madrid 28049 Spain; ^6^ Department of Physiological Chemistry Faculty of Chemistry University of Vienna Vienna 1090 Austria; ^7^ Leibniz Institute for Food Systems Biology Technical University Munich 85354 Munich Germany; ^8^ VetCore Facility for Research University of Veterinary Medicine Vienna 1210 Austria; ^9^ Present address: MacroArray Diagnostics GmbH Vienna 1230 Austria

**Keywords:** allergens, heat stability, meat allergy, myosin light chain, recombinant allergens, red meat, stability to digestion

## Abstract

**Scope:**

Red meat, a staple food of Western diets, can also induce IgE‐mediated allergic reactions. Yet, apart from the heat‐labile protein serum albumin and the carbohydrate α‐Gal, the molecules causing allergic reactions to red meat remain unknown.

**Methods and results:**

IgE reactivity profiles of beef‐sensitized individuals are analyzed by IgE‐immunoblotting with protein extracts from raw and cooked beef. Two IgE‐reactive proteins are identified by peptide mass fingerprinting as myosinlight chain 1 (MYL1) and myosin light chain 3 (MYL3) in cooked beef extract and are designated Bos d 13 isoallergens. MYL1 and MYL3 are produced recombinantly in *Escherichia coli*. ELISAs proved their IgE reactivity and circular dichroism analysis showed that they represent folded molecules with remarkable thermal stability. In vitro gastrointestinal digestion experiments showed the higher stability of rMYL1 as compared to rMYL3. Exposure of a monolayer of Caco–2 cells to rMYL1 indicated that the molecule is able to cross intestinal epithelial cells without disturbing the integrity of the tight junctions, suggesting the sensitizing capacity of MYL1.

**Conclusion:**

MYLs are identified as novel heat‐stable bovine meat allergens.

## Graphical abstract

Extracts are prepared from raw and cooked beef and IgE reactivity profiles of beef‐allergic individuals are analyzed by IgE‐immunoblotting. IgE‐reactive proteins are identified by peptide mass fingerprinting as myosin light chains 1 (MYL1) and 3 (MYL3). Both proteins are produced recombinantly, and their IgE reactivity, secondary structure and digestive stability, including the effect of the food matrix, are investigated. The transport of rMYL1 through the intestinal epithelium is also studied.

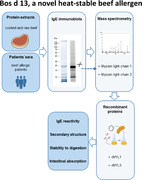

## 1 Introduction

Meat constitutes a staple food in the Western diet. After poultry, the most frequently consumed meats in the Organisation for Economic Co‐operation and Development (OECD) countries are pork and beef, with consumptions of 22.9 and 14.4 kg per capita, respectively.^[^ ^1^ ^]^ Red meat, besides being an essential part of the diet in industrialized countries, has traditionally been regarded as an important source of high‐quality proteins.^[^ ^2^, ^3^ ^]^ However, mammalian meat can also cause allergies.^[^ ^4^ ^]^

Allergies to meat proteins are considered as a rare condition, although in the last decade meat has received an increasing attention as allergen source, mainly due to a special form of allergy to the oligosaccharide α‐Gal, present in mammalian meat.^[^ ^5^ ^]^ Overall, only rough estimates are available, stating that of all food allergies, about 0.5–8% are caused by meat of different origins.^[^ ^6^, ^7^, ^8^ ^]^ In case of mammalian or red meat proteins, reactions have mainly been reported after consumption of beef, with symptoms varying from allergic oral syndrome (OAS) to skin reactions, gastrointestinal symptoms, and even anaphylaxis.^[^ ^9^, ^10^, ^11^ ^]^ Allergy to beef proteins is often associated with allergy to milk, and it has been reported that 20% of children allergic to cow's milk are also allergic to bovine meat.^[^ ^11^, ^12^, ^13^ ^]^ Yet, meat extracts are not included in the routine allergy screening and epidemiologic studies on the prevalence of red meat allergies are missing.^[^ ^14^ ^]^ Furthermore, diagnostic tests are still based on non‐standardized extracts with a very low specificity.^[^ ^15^ ^]^ These commercial extracts are often heterogeneous mixtures that might cause false negative results. This may well have contributed to an underestimation of the incidence of the disease, which, when studied in detail, appears to be more common than previously thought.^[^ ^14^ ^]^

Therefore, the identification and characterization of new red meat allergens, together with their production as recombinant proteins, not only would help to improve the diagnosis of allergy to bovine meat and the understanding of the disease, but it would also open the possibility of treating the condition by immunotherapy using the specific allergens.^[^ ^16^ ^]^

So far, only a few proteins of mammalian origin have been identified as the cause of food allergy reactions.^[^ ^17^, ^18^, ^19^, ^20^, ^21^ ^]^ Of the 11 allergens of bovine origin listed by the WHO/IUIS the majority is solely present in cow´s milk. Beside the oligosaccharide α‐Gal that causes a very unusual, delayed form of red meat allergy,^[^ ^22^ ^]^ only two of the WHO/IUIS listed allergens are present in beef: serum albumin (Bos d 6)^[^ ^23^ ^]^ and bovine immunoglobulin G (Bos d 7).^[^ ^24^ ^]^ Both proteins are also present in cow´s milk and both are heat‐labile proteins^[^ ^25^, ^26^ ^]^ that are at least partially denatured when beef is cooked. Since meat is usually consumed after heat treatment, such as, e.g., boiling or grilling, there is a need for the identification of allergenic beef molecules that resist thermal treatment.

In this study we aimed at identifying heat‐stable red meat allergens. Immunoblots performed with sera from individuals with IgE antibodies to bovine meat followed by peptide mass fingerprinting allowed to identify two variants of myosin light chain: myosin light chain 1 and 3 (MYL1 and MYL3), as IgE reactive molecules in cooked beef. cDNAs coding for the two MYL proteins (designated Bos d 13 isoallergens by the International Union of Immunological Societies allergen nomenclature subcommittee) were cloned from beef skeletal muscle cDNA and were produced as properly folded, recombinant proteins in *Escherichia coli*. ELISA experiments showed that both recombinant proteins represent IgE reactive proteins, containing, at least in case of recombinant myosin light chain 1 (rMYL1), the same IgE binding epitopes as its natural counterpart. Besides its resistance to heat treatment, in vitro digestion experiments indicated that MYL1 is very stable to gastrointestinal digestion and in vitro intestinal transport experiments with Caco‐2 cells showed that recombinant MYL1 can be transported intact across the intestinal epithelium.

In summary, our group has identified the first heat‐stable beef allergens, which, in case of MYL1 can also be an important sensitizer of meat allergy.

## 2 Results

### 2.1 Patients’ igE Reactivity to Raw and Cooked Beef

Extracts of water‐soluble proteins of raw and cooked beef were electrophoretically separated on 12% SDS‐PAGE gels and proteins were either stained with Coomassie Brilliant blue or blotted onto nitrocellulose membranes. The effect of cooking on bovine meat proteins can be seen in the Coomassie stained gel (Figure S1, Supporting Information): most of the proteins of the raw extract, especially those with molecular weights above 35 kDa, were only present in the raw extract, but not in the cooked one. This indicates that many water‐soluble proteins aggregated upon heat treatment and became insoluble in water. Instead, more proteins of lower molecular weight (below 10 kDa) can be seen in the cooked extract, which might represent fragments of heat‐denatured higher molecular weight proteins.

**Figure 1:**
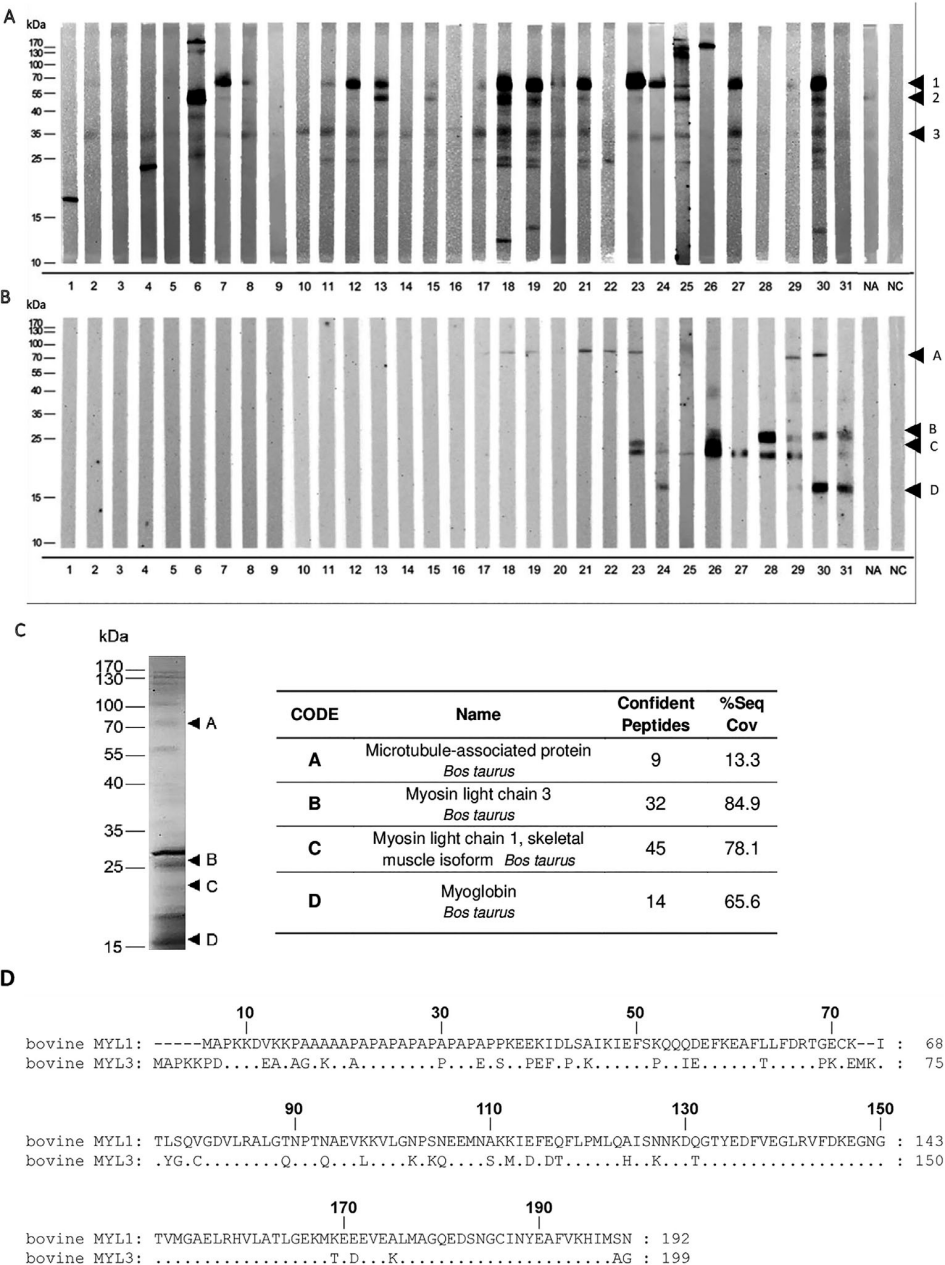
IgE reactivity patterns of patients to bovine meat extract. Extracts from raw (A) and cooked (B) beef separated by SDS‐PAGE blotted onto nitrocellulose membranes and exposed to sera from 31 patients sensitized to bovine meat. Bound IgE antibodies were detected with anti‐human IgE antibodies. Serum of a non‐meat allergic individual (NA) and PBST only (NC) were used as negative controls. Molecular weights (kDa) are indicated in the left margin. IgE reactive bands labeled as 1, 2, and 3 in raw beef and A, B, C, and D in cooked beef. C) Labeled IgE reactive bands were excised from a Coomassie stained gel (left) and the proteins identified by mass spectrometry (right). The number of confident peptides identified of each protein and the percentage sequence coverage are shown. Confident peptides are those peptides that have a 95% probability that they have been assigned correctly to the respective protein. D) Amino acid sequence alignment of bovine myosin light chain 1 (MYL1; UniProt accession number A0JNJ5) and 3 (MYL3; UniProt accession number P85100).

When membranes containing extracts of raw and cooked beef were incubated with sera of 31 patients sensitized to bovine meat (**Table** **1**), different IgE reactivity patterns were observed. Overall, more patients displayed IgE reactivity to proteins present in raw as compared to cooked beef extract. In raw beef immunoblots, the protein most frequently recognized by patients’ IgE antibodies, which also showed strongest IgE reactivity, was a protein of 70 kDa. Sixteen out of 31 patients (7, 11, 12, 13, 15, 17, 18, 19, 20, 21, 23, 24, 25, 27, 29, and 30) showed IgE reactivity to this protein (**Figure** **1**A, protein 1). In addition, a 55 kDa protein (Figure 1A, protein 2) was recognized by IgE antibodies of eight patients (6, 13, 15, 18, 19, 21, 25, and 30). All the other IgE reactive proteins were either only recognized by one or two patients (e.g., proteins of 12, 15, 17, 25, 130, 170 kDa) or they displayed very low IgE reactivity (e.g., proteins of 30 and 35 kDa). In case of the 35 kDa protein (Figure 1A, protein 3), this was also weakly recognized in a control immunoblot performed with the serum from a non‐meat allergic individual (NA), suggesting that it does not represent a molecule specifically recognized by IgE antibodies of meat allergic sensitized patients.

**Table 1:** Demographic, clinical, and serological characteristics of patients with IgE antibodies to beef

Patient	Sex	Age [y]	Medical records	IgE [kUA l^−1^]			
				Total	Beef	Pork	Lamb
1	F	26	Anaphylaxis to cow milk, beef and pork meat	95	2.59	0.52	2.13
2	M	19	CMPA	184	0.86	‐	‐
3	M	32	Subclinic sensitization to cow milk	1369	0.42	1.45	‐
4	M	9	outgrown CMPA	92.6	0.63	0.93	‐
5	F	4	Abdominal pain after eating pork, CMPA	124	1.3	0.02	‐
6	M	7	Angioedema with beef, outgrown CMPA	92.5	2.01	‐	‐
7	M	3	CMPA	186	1.63	‐	‐
8	F	3	Perioral urticaria with beef, CMPA	1402	1.56	‐	‐
9	M	6	Perioral urticaria with lamb meat	122	1.45	0.13	1.02
10	M	2	OAS after eating meat products, CMPA	72.7	0.74	‐	‐
11	F	2	Diarrhea after eating beef, CMPA	129	1.15	‐	‐
12	M	3	Perioral urticaria with beef, CMPA	34.5	1.7	2.83	0.99
13	M	2	CMPA	13.4	0.82	‐	‐
14	F	3	Urticaria after eating beef, pork and Lamb meat, CMPA	167	3.22	4.29	1.24
15	M	2	CMPA	14.9	0.92	‐	0.22
16	M	7	Eosinophilic esophagitis	229	0.53	‐	‐
17	M	3	Vomiting after eating beef, CMPA	67.6	9.4	‐	‐
18	F	6	CMPA	24.4	3.71	‐	‐
19	F	1	CMPA	72	9.48	‐	‐
20	M	13	CMPA	392	1.94	‐	‐
21	M	15	OAS after eating lamb. Positive prick test to beef, CMPA	430	4.25	3.05	‐
22	M	2	CMPA	114	36.7	‐	‐
23	M	1	CMPA	625	5.13	‐	‐
24	M	4	OAS with beef, CMPA	251	0.92	‐	‐
25	M	13	Urticaria after eating beef, CMPA	1913	2.36	1.31	1.96
26	M	7	OAS with beef	2052	2.15	‐	‐
27	F	2	OAS with beef, CMPA	74.7	1.09	‐	‐
28	M	33	Eosinophilic esophagitis	557	0.64	‐	‐
29	M	3	Vomiting after eating beef, CMPA	984	14.9	14.2	‐
30	M	2	Positive skin prick test for cow, outgrown egg allergy	500	4.25	3.05	‐
31	M	13	CMPA	818	1.23	‐	‐

**CMPA,** cow's milk protein allergy; **kUA/l**, kilo units of allergen per liter as determined by **ImmunoCAP** (Thermo Fisher, Uppsala, Sweden); **F**, female; **M**, male; **y**, years; ‐, not tested.

In contrast, none of the proteins displaying IgE reactivity in the raw extract was recognized by patients’ IgEs in the cooked beef extract (Figure 1B). Surprisingly, most of the patients did not show any IgE binding to proteins present in cooked beef. However, nine patients (23–31) exhibited rather strong IgE binding to proteins between 20 and 25 kDa (Figure 1B, proteins B and C) and four of them (24, 29, 30, and 31) also recognized a protein of around 15 kDa in the cooked extract (Figure 1B, protein D). In addition, eight patients (17, 18, 19, 21, 22, 23, 29, and 30) displayed rather weak IgE reactivity to a protein of ≈100 kDa (Figure 1B, protein A). In control experiments performed with the serum from an individual not sensitized to meat (NA) and with buffer only (NC) no reactivity was observed.

### 2.2 Identification of Myosin Light Chain as an IgE Reactive Protein in Cooked Beef by Mass Spectrometry

Protein bands recognized by patients’ IgE antibodies in the raw and cooked beef extracts (Figure 1) were excised from the Coomassie‐stained gel (Figure 1C), subjected to trypsin digestion, and analyzed by LC‐ESI‐MS/MS mass spectrometry for identification. In the raw beef extract, as expected, the protein of 70 kDa, recognized by 16 of the patients (Figure 1A, protein 1), was identified as the major beef allergen bovine serum albumin. Serum albumin is known as a thermolabile protein and indeed, the 70 kDa protein was only recognized by patients’ IgE antibodies in the raw beef extract (Figure 1A), but not in the cooked beef extract (Figure 1B). No protein could be unambiguously identified by mass spectrometry in case of the 55 kDa protein band (Figure 1A, protein 2). The 35 kDa protein weakly recognized by most of the patients (Figure 1A, protein 3), was identified as tropomyosin. Tropomyosin is a well‐known invertebrate pan allergen^[^ ^27^ ^]^ and it had been reported that a minority of meat‐allergic patients recognized it weakly.^[^ ^21^ ^]^ In our study, not only did it show a weak IgE reactivity among the red meat patients, but it was also recognized by the individual not sensitized to meat.

In the cooked beef extract, the IgE reactive bands between 20 and 25 kDa (bands C and B in Figure 1B) were identified as bovine myosin light chain 1/3 (MYL1) and bovine myosin light chain 3 (MYL3), with 78.1% and 84.9% of sequence coverage respectively. Table S1, Supporting Information, lists the identified peptides and shows that some peptides can be assigned to both proteins, MYL1 and MYL3. The IgE reactive band of approximately 15 kDa (band D in Figure 1B) was identified as myoglobin with a sequence coverage of 65.5% and the band of about 100 kDa (band A in Figure 1B) was identified as microtubule‐associated protein, but with a very low sequence coverage of 13.3% (Figure 1C).

Because the proteins between 20 and 25 kDa displayed the strongest IgE reactivity in cooked beef we decided to focus our investigations on the two IgE‐reactive myosin light chain proteins: MYL1 (accession number A0JNJ5) and MYL3 (accession number P85100). Since the two proteins have a similar molecular size (20.939 kDa MYL1 and 21.939 kDa MYL3), with a 76% homology of their amino acid sequences (Figure 1D), and demonstrate similar biological functions, they were officially named as isoallergens Bos d 13.0101 and Bos d 13.0201 by the World Health Organization and International Union of Immunological Societies (WHO/IUIS) Allergen Nomenclature Sub‐committee and are listed in the official allergen database (www.allergen.org).^[^ ^28^ ^]^

### 2.3 Generation of Recombinant Bovine MYL1 and MYL3 as Properly Folded, IgE Reactive Proteins

The two myosin light chain isoallergens MYL1 and MYL3 were produced as recombinant proteins in *E. coli*. For this, sequences coding for bovine MYL1 and MYL3 were amplified by PCR from cow muscle cDNA and then cloned into the expression vector pET‐17b with a C‐terminal hexahistidine tag. Both proteins were expressed as soluble proteins and were purified by immobilized metal affinity chromatography performed under native conditions. A Coomassie stained SDS‐PAGE gel showed that the recombinant proteins had the expected molecular weight of approximately 21 kDa (rMYL1) and 22 kDa (rMYL3) and indicated that they both represent pure proteins (Figure S2, Supporting Information).

**Figure 2:**
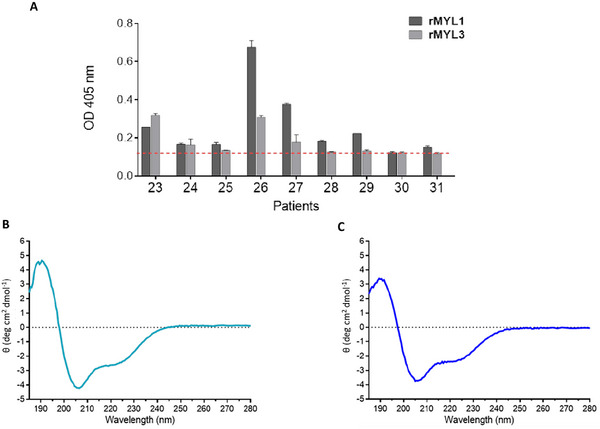
A) IgE binding capacity of rMYL1 and rMYL3. IgE binding to rMYL1 and rMYL3 was determined by ELISA. ELISA plate‐bound rMYL1 and rMYL3 were incubated with sera of beef sensitized patients (23–31). Mean OD values corresponding to IgE binding were measured by ELISA. The dashed line represents the mean value of the negative controls plus threefold the standard deviation. **B** and **C**) rMYL1 and rMYL3 represent folded proteins with mainly alpha‐helical secondary structure. Far‐UV circular dichroism analysis was performed with rMYL1 and rMYL3 in a wavelength range from 190 to 280 nm. The spectra are expressed as molar circular dichroism *θ* (deg cm^2^ dmol^−1^) at a given wavelength.

For evaluation of the IgE reactivity of rMYL1 and rMYL3 an ELISA was performed with sera (patients 23–31) that had recognized the 20 kDa and/or the 25 kDa protein in the IgE immunoblot of cooked beef extract (Figure 1B). From the nine patients included all, but patient 30, showed IgE reactivity to rMYL1 (**Figure** **2**A), whereas IgEs of only four patients (patients 23, 24, 26, and 27) recognized rMYL3.

Since the IgE reactivity of both recombinant molecules was rather low (OD values below 0.6), we investigated by circular dichroism (CD) spectrometry whether they were properly folded (Figure 2B,C). The far‐UV spectra of rMYL1 and rMYL3 recorded at room temperature are characterized by two minima at around 207 and 220 nm and a maximum at 193 nm. Such spectra are typical for folded proteins with mainly α‐helical secondary structure. A further analysis using the web server BeStSel^[^ ^29^ ^]^ for prediction of secondary structure from the circular dichroism spectra indicated that 25.8% of the rMYL1 and 21.1% of the rMYL3 structure were α‐helices, whereas 17.5% of the rMYL1 and 15.7% of the rMYL3 structure were antiparallel β‐sheet structures. The dominance of α‐helices in the structure of rMYL1 and rMYL3 was in accordance with the secondary structure predicted from the amino acid sequences of the proteins using the PSIPRED server.^[^ ^30^ ^]^ These results proved the proper folding of the two recombinant allergens.

### 2.4 rMYL1 and rMYL3 Show High Thermal Stability and they Partially Resists Gastric but Not Duodenal Digestion

To investigate the thermal stability of rMYL1 and rMYL3, their thermal denaturation and refolding capacity were followed by CD spectroscopy during gradual increasing of the temperature to 90 °C and re‐cooling to 20 °C (Figure S3, Supporting Information). In case of both proteins, an increase of the temperature resulted in less pronounced minima at 207 and 220 nm. However, even at the highest temperature of 90 °C the minima were only slightly shifted to shorter wavelengths (Figure S3A,B, Supporting Information). Furthermore, both proteins were able to fold back to their original secondary structure upon cooling (Figure S3C,D, Supporting Information), indicating the high thermal stability of both proteins.

**Figure 3:**
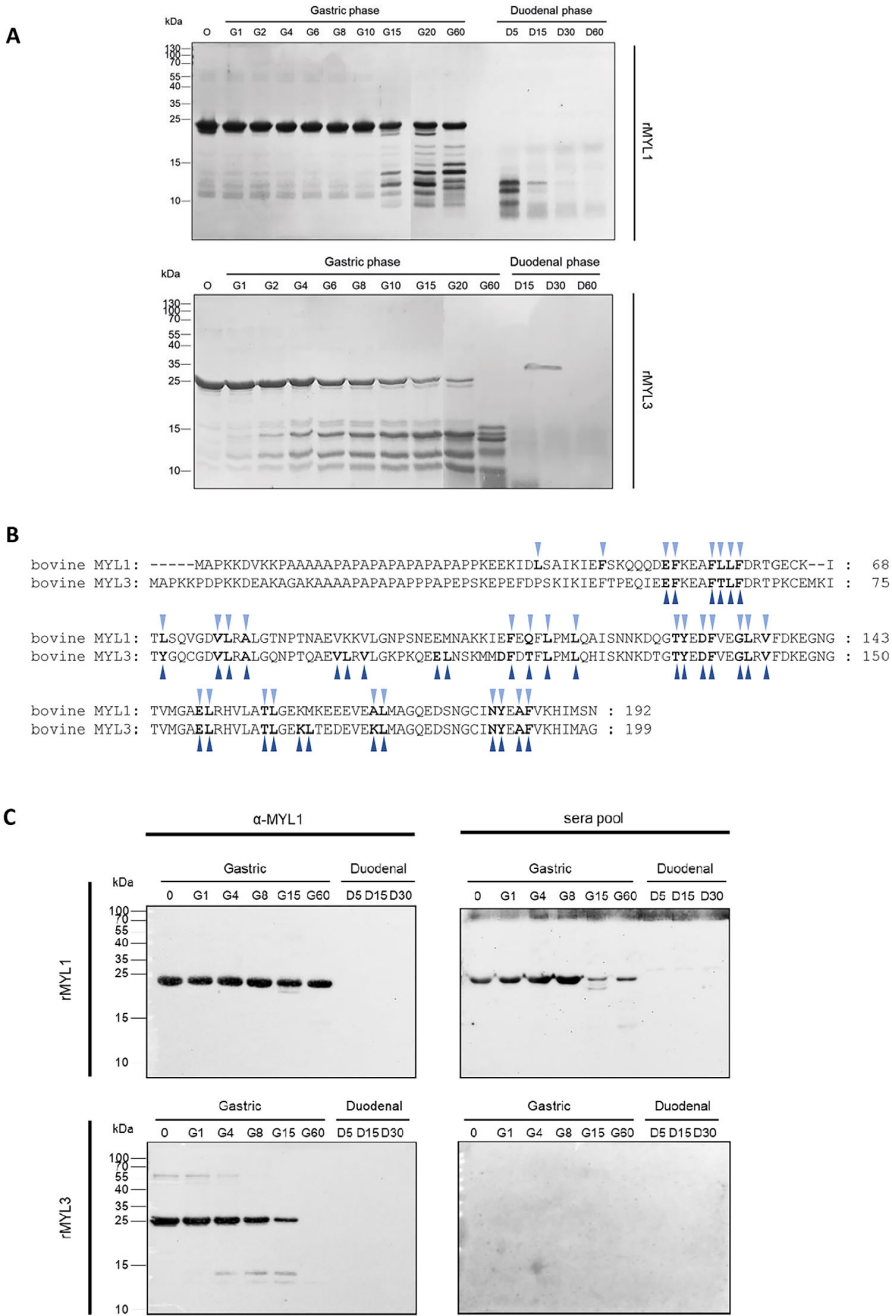
Effect of simulated gastric and duodenal digestion on the stability of rMYL1 and rMYL3. **A**) Coomassie stained SDS‐PAGE of the aliquots taken during in vitro digestion of rMYL1 (upper panel) and rMYL3 (lower panel) before being exposed to proteolytic enzymes (0) and after being exposed to pepsin for 1, 2, 4, 6, 8, 10, 15, 20, and 60 min (G1, G2, G4, G6, G8, G10, G15, G20, G60) and subsequently to trypsin and chymotrypsin for 5, 15, 30, and 60 min (D5, D15, D30, D60). B) Pepsin (gastric phase) cleavage sites identified by the ExPASy – PeptideCutter in the amino acid sequences of MYL1 (light blue triangles) and MYL3 (dark blue triangles). C) Aliquots 0, G1, G4, G8, G15, G60, D5, D15, and D30 of in vitro digestions of rMYL1 and rMYL3 were blotted onto nitrocellulose and either incubated with the rabbit anti‐rMYL1 antiserum (left) or with a pool of patients´ sera (23–27) (right). Molecular weights (kDa) are always indicated in the left margin.

Since it is known that allergens sensitizing via the gastrointestinal tract need to be resistant to gastrointestinal digestion, the stability of rMYL1 and rMYL3 was analyzed in in vitro digestion experiments following the method by Moreno et al.^[^ ^31^ ^]^ Aliquots taken at different time points during the simulated gastric and duodenal digestion, were analyzed by SDS‐PAGE and immunoblotting. The Coomassie stained SDS‐PAGE gel in **Figure** **3**A shows the higher stability of rMYL1 as compared to rMYL3 to gastric digestion. In case of rMYL1, first degradation products (at molecular weights of 12–13 kDa) only appear after 15 min of peptic digestion (G15), and even after 60 min of gastric digestion, the majority of rMYL1 was still intact and appeared at a molecular weight of 22 kDa (G60). On the other hand, first degradation products of rMYL3 (at molecular weights of 12 and 14 kDa) were already visible 1–2 min after initiation of gastric digestion (G1 and G2). In the aliquot taken after 60 min of peptic digestion the protein band at 23 kDa corresponding to intact rMYL3 was no longer visible (G60). Analysis of the theoretical pepsin cleavage sites in the protein sequence of MYL1 and MYL3 using the protease digestibility prediction tool ExPASy – PeptideCutter shows that MYL1 contains 33 potential cleavage sites for pepsin, whereas MYL3 has 39 (Figure 3B).

After addition of trypsin and chymotrypsin to the in vitro digestion, however, rMYL1 was also quickly digested and already after 5 min of simulated duodenal digestion no intact rMYL1 protein was any more visible on the gel (D5).

Immunoblots performed with a rabbit antiserum generated against rMYL1 that recognized rMYL1 and rMYL3 confirmed the SDS‐PAGE results (Figure 3C). The anti‐rMYL1 antiserum (α‐MYL1) detected the intact rMYL1 protein in all the samples taken from the peptic digest up to 60 min of digestion (G1–G60), whereas intact rMYL3 was only detected by the antibody up to 15 min of gastric digestion (G1–G15 in Figure 3C). Whereas degradation products of rMYL3 were detected by the anti‐rMYL1 antiserum at molecular weights of 13–14 kDa in G4, G8, and G15, none of the degradation products of MYL1 was recognized by the anti‐rMYL1 antiserum. In those samples taken from the duodenal digestions (D5, D15, D30 in Figure 3C), neither rMYL1 nor rMYL3 nor their degradation products were detected by the anti‐rMYL1 antiserum. To see whether the intact rMYL1 and rMYL3 and their digestion products still showed IgE reactivity, immunoblots were performed with the gastrointestinal digestion samples using a pool of four patients’ sera (patients 23, 24, 26, and 27). Patients’ IgE antibodies recognized intact rMYL1 in all the aliquots of the gastric digestion (G1–G60 in Figure 3C), but not in the aliquots taken from the simulated duodenal digestion (D5–D30). Interestingly, none of the fragments generated during gastrointestinal digestion displayed IgE binding capacity. In case of rMYL3 it was not possible to determine whether IgE reactivity was retained, because also the intact protein in the sample taken before addition of pepsin (G0) was not recognized by the serum pool, even though the sera of the four patients had shown weak IgE reactivity to rMYL3 in the ELISA (Figure 2C).

This lack of reactivity could be due to the fact that the IgE antibodies were much more diluted in the immunoblot than they were in the ELISA. To perform the ELISA, the serum of each individual was tested separately and diluted 1:5. In contrast, for the immunoblot, a pool of four sera was used. For preparation of the pool, equal volumes of each serum were first mixed, and then the pool was further diluted 1:10, causing a final dilution of 1:40 of each individual serum.

### 2.5 Food Matrix Protects MYL1 from Pancreatic Enzymes’ Digestion

**Figure 4:**
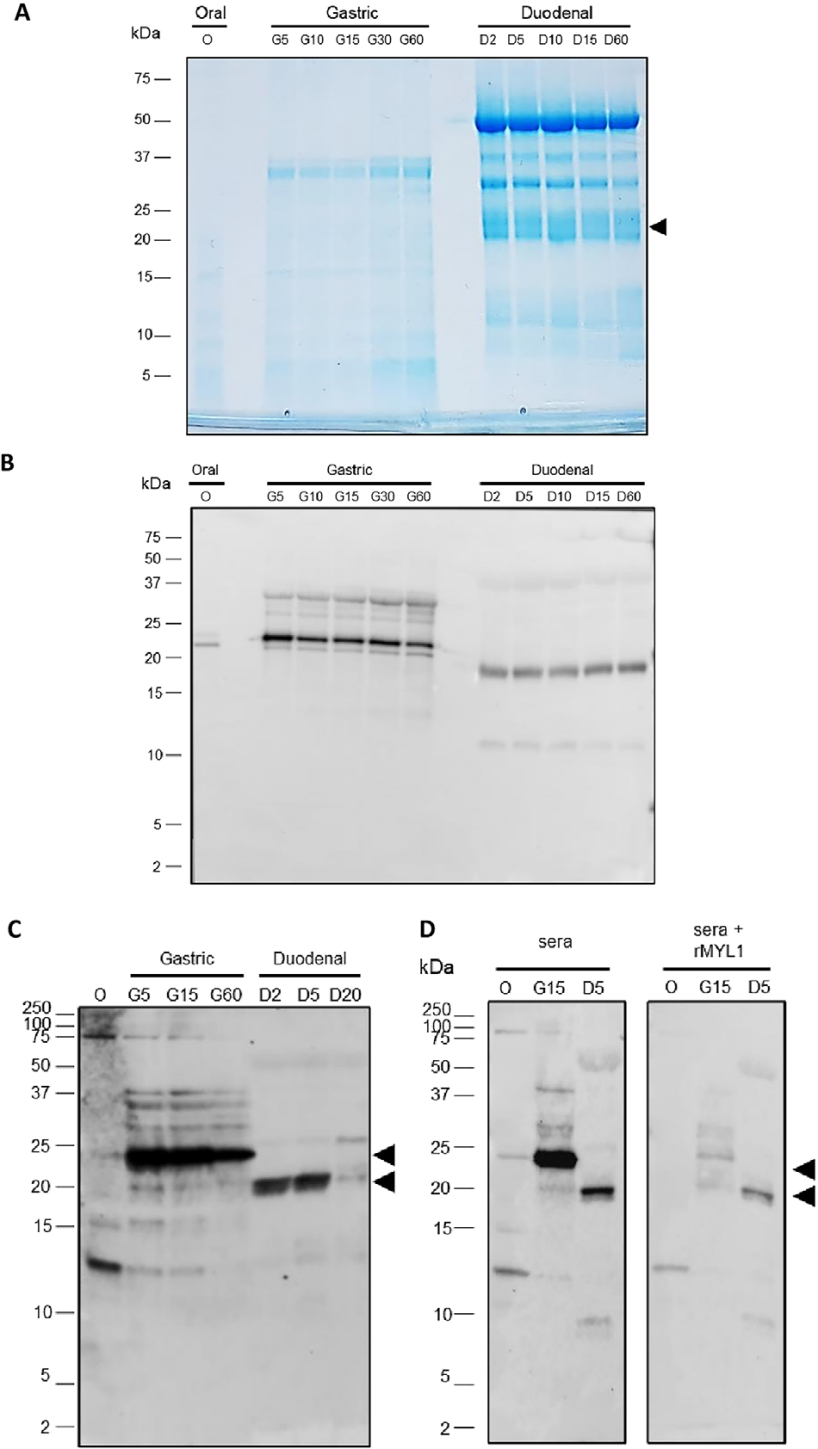
The impact of food matrix on the stability of MYL1 and/or MYL3. A) Coomassie stained Tris‐tricine SDS‐PAGE gel of the aliquots taken during the oral phase of the in vitro digestion of cooked beef (O), and after 5, 10, 15, 30, and 60 min of simulated gastric digestion (G5, G10, G15, G30, G60) and 2, 5, 10, 15, and 60 min of simulated in vitro intestinal digestion (D2, D5, D10, D15, D60). B) Aliquots O, G5, G10, G15, G30, G60, D2, D5, D10, D15, and D60 of the in vitro digestions were blotted onto a nitrocellulose membrane and exposed to the anti‐rMYL1 antiserum. C) Aliquots O, G5, G15, G60, D2, D5, and D20 were blotted onto nitrocellulose and incubated with a pool of patients´ sera (patients 23, 24, 26, and 27). D) Aliquots 0, G15, and D5 were blotted onto a nitrocellulose membrane and then exposed to a pool of sera from patients 23, 24, 26, and 27 which was either pre‐incubated with PBS (sera) or with rMYL1 (sera+rMYL1). Molecular weights (kDa) are always indicated in the left margin. The molecular weight of MYL1 is indicated with arrows.

To investigate the impact of food matrix on the digestion process of natural MYL, a piece of cooked beef was subjected to in vitro digestion following the INFOGEST consensus method described by Minekus et al.^[^ ^32^ ^]^ For this, the cooked beef was minced and resuspended in simulated salivary fluid (O) and then exposed to simulated gastric fluid (SGF) and to simulated intestinal fluid (SIF) for 60 min each. Aliquots taken after 5, 10, 15, 30, and 60 min (G5, G10, G15, G30, G60) of gastric digestion and 2, 5, 10, 15, 20, and 60 min (D2, D5, D10, D15, D20, D60) of duodenal digestion were first analyzed on a Coomassie stained Tris‐tricine gel (**Figure** **4**A). After 2 min in the simulated salivary fluid of the oral phase (O), not many beef proteins were yet solubilized. Therefore, only a few weak bands with sizes below 25 kDa are visible. In the gastric phase, the low pH of the SGF and the presence of pepsin contributed to the solubilization of further proteins. Therefore, more protein bands together with a smear, which suggests protein digestion (Figure 4A), can be seen in the samples of the gastric digestion (G5–G60). The distinct protein band at 37 kDa represents the added pepsin. The progression of the digestion is seen by the increasing intensity of the bands in the lower molecular weight, especially those between 10 and 15 kDa in the aliquots taken at later time points of gastric digestion (G30, G60) and during the duodenal digestion (D10, D15, D60). In the duodenal digestion, strong bands are visible at molecular weights of ≈23, ≈25, and ≈50 kDa, which correspond to the molecular weights of digestive enzymes present in pancreatin: trypsin, chymotrypsin, and lipase, respectively. Bands at ≈30 and ≈37 kDa in the duodenal digestion correspond most likely to lipase and pepsin and degradation products thereof. The molecular weight of myosin light chain at ≈22 kDa is marked in the gel with a black arrow.

To see whether MYL is present in the aliquots of digested cow's meat, immunoblots were performed with the anti‐rMYL1 rabbit antiserum (Figure 4B). The anti‐rMYL1 antiserum recognized MYL as a protein band of ≈22 kDa in the oral phase. The weakness of the signal is due to the fact that in the oral phase only small amounts of MYL were solubilized. In the aliquots taken from the gastric digestion (G5–G60) and, remarkably, also in the aliquots from the duodenal digestion (D2–D60) MYL was detected by the antibody (Figure 4B). The amount of protein detected slightly decreased with the progression of the gastric digestion. When the intensities of the signals were analyzed using ImageJ software (National Institutes of Health, Bethesda, MD, USA), the intensity of the signal in G60 was 48.7% lower than the intensity of the signal in G5. Interestingly, the protein band recognized by the antibody in all the aliquots from the duodenal digestion showed a lower molecular weight (≈20 kDa) than the protein detected in the oral and gastric phases (≈22 kDa). This lower molecular weight could be due to the partial hydrolyzation of MYL by pancreatic enzymes (Figure 4B).

To investigate whether MYL and the fragments generated during digestion display IgE reactivity, IgE immunoblots were performed with the different aliquots (O, G5, G15, G60, D2, D5, D20) and with a pool of patients' sera (patients 23, 24, 26, and 27 in Figure 4C). In the oral phase aliquot (O) MYL was only detected as a faint 22 kDa protein band by patients´ IgE antibodies. This is due to the low amount of MYL that was solubilized in the oral phase. However, the serum pool showed strong IgE reactivity to MYL in the aliquots of the gastric digest (G5, G15, and G60) and in the aliquots D2 and D5 of the duodenal digest. Interestingly, in D20 only a very weak IgE‐reactive protein band is visible, pointing to a destruction of IgE binding epitopes during longer duodenal digestion. Comparable to the results obtained with the anti‐rMYL1 antiserum (Figure 4B), MYL appears at a molecular weight of 22 kDa in the gastric digest and at 20 kDa in the duodenal digest, suggesting partial digestion of MYL by pancreatic enzymes in the samples from the duodenal digest. To confirm that the IgE reactive bands of 22 and 20 kDa are indeed MYL, an IgE inhibition immunoblot was performed. For this, the serum pool was preincubated overnight with rMYL1 and then the nitrocellulose blotted digestion aliquots O, G15, and D5 were exposed to the preincubated serum pool. As can be seen in Figure 4D, rMYL1 significantly reduced patients’ IgE binding to the 22 kDa band in O and G15, confirming that the protein indeed represents MYL1. Preincubation of the sera with rMYL1 also decreased IgE binding to the protein band of 20 kDa in the duodenal aliquot D5, indicating that the 20 kDa protein band represents an IgE reactive hydrolysis product of MYL1 (Figure 4D).

### 2.6 Recombinant MYL1 Is Transported Across the Gastrointestinal Epithelium

**Figure 5:**
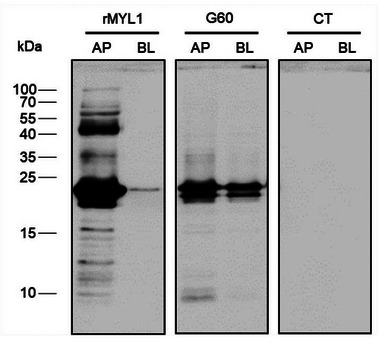
rMYL1 is transported intact through a monolayer of Caco‐2 cells. Anti‐rMYL1 immunoblot of the medium applied to the apical (AP) side and collected from the basolateral side (BL) of a Caco‐2 cell monolayer cultured on permeable supports. Cells were incubated with undigested rMYL1, with rMYL1 digested by pepsin for 60 min (G60) or with medium (CT) only. Molecular weights (kDa) are indicated in the left margin.

In order to cause systemic reactions, food allergens need to cross the intestinal epithelium. We, therefore, investigated in an in vitro system, whether rMYL1 can be transported intact across a monolayer of enterocytes. Caco‐2 cells were used as a model for enterocytes and were grown on permeable inserts for 21 days until the cells were fully differentiated, and a tight monolayer was formed (TEER >400 Ω cm^−2^). Then, undigested rMYL1 and rMYL1 after 60 min of gastric digestion (G60) were added to the apical (AP) side of the Caco‐2 cells. On the next day, TEER measurements proved that the integrity of the cell monolayer was not affected by incubation with the allergen (TEER values were still >400 Ω cm^−2^). Then, the media from the apical (AP) and basolateral (BL) side were collected and were used for detection of rMYL1 in immunoblots using the rabbit anti‐MYL1 polyclonal antibody (**Figure** **5**). In the experiment performed with the undigested sample, the majority of rMYL1 was found in the apical medium (AP). However, small amounts of rMYL1 were also detected in the medium of the basolateral chamber, indicating that rMYL1 can be transported intact across the Caco‐2 cell monolayer. Interestingly, when digested rMYL1 (G60) was added to the apical side of the Caco‐2 cell monolayer, higher amounts of rMYL1 were transported through the cells into the basolateral medium after overnight incubation (Figure 5).

### 2.7 Bovine Myosin Light Chain Cross‐Reacts with Porcine Myosin Light Chain

**Figure 6:**
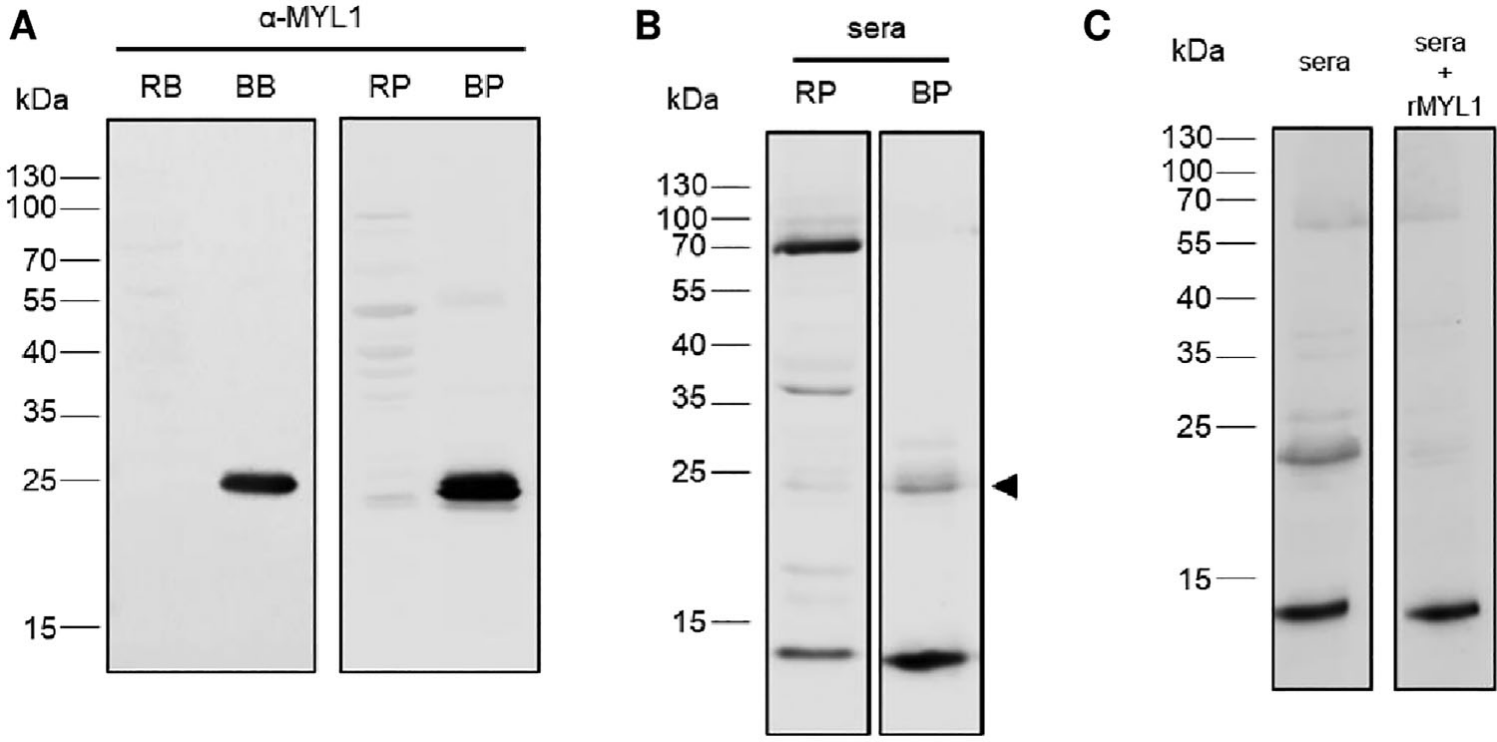
Bos d 13 cross‐reacts with pig myosin light chain. A) Anti‐rMYL1 immunoblot of raw (RP) and boiled (BP) pork. B) IgE reactivity of a pool of sera from sensitized patients incubated with blotted proteins of raw pork (RP) or boiled pork (BP). C) Immunoblot of boiled pork proteins incubated with a serum pool from beef sensitized patients (23, 25, 26, and 29) which was either pre‐incubated with buffer (sera) or with rMYL1 (sera+rMYL1). Molecular weights (kDa) are always indicated in the left margin. The molecular weight of MYL1 is indicated with an arrow.

Amino acid sequences of bovine and porcine myosin light chain 1 show a 99% identity (data not shown). To analyze the cross‐reactivity between beef and porcine myosin light chain proteins, it was first investigated, whether the rabbit serum generated against rMYL‐1 would recognize porcine myosin light chain. For this, protein extracts from raw and cooked pork and, for control purposes, also from beef were blotted onto a nitrocellulose membrane and were incubated with the anti‐rMYL1 antibody. Myosin light chain was neither detected in raw beef nor in raw pork extracts. These results corroborated our IgE immunoblots (Figure 1A), where myosin light chains were also not bound by patients’ IgE antibodies in the raw extract. In a previous study on chicken myosin light chain (Gal d 7) we also saw that Gal d 7 was not present in raw protein extracts and that cooking or denaturing conditions were required for solubilisation of Gal d 7.^[33]^ However, the anti‐rMYL1 antibody recognized a ≈22 kDa protein in both, cooked bovine and porcine extract (**Figure** **6**A). To evaluate patients’ IgE reactivity to porcine myosin light chain proteins, blotted raw and cooked pork meat proteins were exposed to a pool of sera (patients 23, 25, 26, and 29, sensitized to beef and IgE‐reactive to bovine rMYL1). Patients’ IgEs bound to proteins of different molecular weights in the raw pork extract (12, 36, and 70 kDa) (Figure 6B), and to proteins of 12 and 22 kDa in the cooked pork extract. Based on the molecular weight, the latter could represent myosin light chain (Figure 6B). To confirm that the 22 kDa protein represents indeed a myosin light chain protein and to evaluate its IgE cross‐reactivity with bovine MYL1, the pool of patients’ sera was preincubated with bovine rMYL1 prior to incubation with blotted proteins of cooked pork (Figure 6C). Binding of IgE antibodies to the 22 kDa pork protein was indeed inhibited by preincubation of the sera with rMYL1, indicating IgE cross‐reactivity between bovine and porcine myosin light chain 1 allergens.

## 3 Discussion

In this study we identified and characterized the first two heat‐stable allergenic proteins from beef, namely myosin light chain 1 and 3. The protein bands in cooked beef immunoblots recognized by IgEs from beef sensitized patients were analyzed by peptide mass fingerprinting and two proteins between 20 and 25 kDa were identified as myosin light chain variants 1 and 3, respectively. After expression of these two myosin light chains as recombinant proteins in *E. coli*, the IgE reactivity of recombinant MYL1 and MYL3 was assessed by ELISA, which showed that both recombinant proteins, rMYL1 and rMYL3, represent IgE reactive proteins. However, MYL1 was recognized by more patients (8) than MYL3 (4).

Skeletal muscle myosin is a hexameric protein consisting of two heavy chains (230 kDa) and four light chains: two regulatory light chains and two essential or alkali light chains.^[^ ^33^ ^]^ The later are involved in actin binding.^[^ ^34^ ^]^ Together, myosin heavy chains and myosin light chains form the myosin protein complex, which is involved in many different processes such as muscle contraction, cytokinesis, cell adhesion, and cell migration. Myosin light chains are required for proper formation and maintenance of the myosin complex.^[^ ^35^, ^36^ ^]^ Myosin light chains belong to a superfamily of calcium‐binding proteins that are characterized by highly conserved helix‐loop‐helix calcium‐binding domains (named EF hands).^[^ ^35^, ^36^ ^]^ Since it is known that the IgE binding capacity of other EF hand calcium binding allergens depends on the presence of calcium,^[^ ^37^ ^]^ we analyzed in an ELISA the effect of calcium on the IgE binding capacity of rMYL1 and saw that neither the presence nor the absence of calcium had an effect on the molecule's IgE binding capacity (data not shown). Our results indicate that the IgE reactivity of EF hand calcium binding allergens is not always calcium dependent.

Whereas MYL1 (20.932 kDa) is expressed in fast twitch muscle fibers, MYL3 (21.939 kDa) is expressed in slow twitch fibers of skeletal and cardiac muscle.^[^ ^38^ ^]^ Various isoforms of myosin alkali light chain have been described. They are encoded by the myosin light chain gene family and each of them is associated with a different muscle type. Fast twitch muscle fibers are more abundant in muscles like the Musculus longissimus dorsi or Musculus semimembr**ano**sus. From these muscles the more commonly eaten cuts of beef, such as the rib eye, top loin, and the chuck eye or rump steak are obtained. Slow twitch fibers are more abundant in precision muscles, like the Musculus psoas major, which is the cut known as tenderloin which is not so frequently consumed.^[^ ^39^, ^40^ ^]^ Individuals would therefore be more exposed to MYL1, present in fast twitch muscle fibers than to MYL3, which makes the allergic sensitization to MYL1 more likely and might explain the lower IgE reactivity and the smaller number of patients reacting to MYL3.

Surprisingly, analysis of the IgE reactivity of the recombinant myosin light chain proteins by ELISA showed that IgE reactivity of rMYL1 was lower than expected. IgE reactivity was also low in the case of patients' sera that showed intense IgE reactivity to natural myosin light chain proteins (Figure 1) in the immunoblots. One possible explanation could be that IgE binding epitopes of food allergens are usually linear epitopes and only rarely conformational epitopes, which would be denatured during digestion.^[^ ^41^ ^]^ However, in an ELISA, the proteins are in their three‐dimensional conformation and therefore, the linear IgE‐binding epitopes are not as accessible as they are in the blot, where proteins are denatured and IgE epitopes linearized. An IgE immunoblot, which showed strong IgE reactivity of rMYL1 and weaker, but still clearly visible, reactivity of rMYL3 supported our hypothesis (Figure S4, Supporting Information).

Our group previously identified myosin light chain 1 (Gal d 7) and myosin light chain 3 as major allergens in cooked chicken meat.^[^ ^42^ ^]^ Here, we showed that myosin light chains also represent allergens in cooked bovine meat. Moreover, IgE antibodies against bovine MYL1 cross‐react with myosin light chain in pork. Although patients sensitized to red meat allergens are usually not reacting to poultry and vice versa, the amino acid sequences of chicken and beef myosin light chain 1 share 87% amino acid identity and chicken myosin light chain 1 (Gal d 7) and beef MYL3 amino acid sequences are 81% homologous; therefore, it would be of interest to investigate the possible cross‐reactivity between these two proteins.

However, and in contrast to chicken, myosin light chains can only be regarded as minor allergens in beef meat. Considering the more than 90% sequence identity between bovine and human myosin light chains it is anyway remarkable that individuals produce IgE antibodies against myosin light chain proteins from beef that do not recognize the human proteins and would thus represent autoantibodies. The high similarity between bovine and human myosin light chains leaves little room for the presence of bovine myosin light chain specific IgE epitopes. It will in future be very interesting to identify these IgE binding epitopes. Based on the results of the IgE immunoblots performed with cooked beef and sera from 31 patients sensitized to bovine meat, it can be concluded that no protein in cooked beef fulfills the criteria of a major allergen, since none of the proteins is recognized by more than 50% of beef sensitized individuals (Figure 1). Intriguingly, many of the beef sensitized patients do not display IgE reactivity to any protein, especially in cooked beef (Figure 1). One possible explanation for this could be that the majority of our patients is, in fact, primarily sensitized to cow's milk because, due to the lack of awareness about this form of allergy, patients are not routinely screened for the presence of IgE antibodies to mammalian meat.^[^ ^14^ ^]^ Another possible reason is that commercial extracts used for diagnosis are prepared from raw meat. Therefore, most of the patients included in our study based on their IgE reactivity to cow's meat were actually sensitized to bovine serum albumin, which is present in milk and in meat only when it is raw, but they are not necessarily sensitized to proteins in beef once it is cooked. That said, we would suggest that commercial extracts used in testing should also contain cooked meat.

Serum albumin is regarded as a major allergen in beef^[^ ^12^ ^]^ and also in our experiments, probably for the reasons discussed above, serum albumin is the protein most frequently recognized by the patients in raw beef extracts (Figure 1). Keeping in mind that meat is normally consumed heat treated, the importance of serum albumin as meat allergen is questionable. Serum albumins are heat‐labile proteins and, as shown already by others, cooking significantly reduces their allergenicity.^[^ ^25^ ^]^

Therefore, the aim of this study was to identify meat allergens that would resist thermal processing of meat. During cooking, proteins might undergo significant modifications. Cooking can also have an influence on the stability of proteins during digestion and on the way they are taken up by intestinal cells.^[^ ^43^, ^44^, ^45^ ^]^ Heat can also induce the formation of Maillard reaction products between amino acids and reducing sugars^[^ ^43^, ^46^ ^]^ and cause denaturation and unfolding of the proteins, resulting in a loss of their secondary and tertiary structure, which can lead to the formation of insoluble aggregates or to the exposure of previously hidden IgE epitopes. In the case of bovine myosin light chain, comparable to our previous findings in chicken meat,^[^ ^42^ ^]^ patients recognized the myosin light chain in cooked but not in raw meat. The reason for this might be that, at temperatures of about 40 °C, myosin light chains dissociate from the myosin hexamer complex, solubilize and previously hidden IgE epitopes become exposed. The heavy chains instead, become insoluble.^[^ ^47^ ^]^ Since heat‐induced denaturation of proteins can also cause the formation of insoluble aggregates, we are aware that one limitation of our study is that we did not analyze insoluble proteins and could thus have missed insoluble IgE‐reactive allergens. The reason for the focus on soluble proteins was that almost all allergens identified so far are water‐soluble. However, it would certainly be of interest to evaluate in the future also the IgE‐reactivity of beef sensitized patients to insoluble bovine meat proteins extracted using a denaturing buffer.

One of the key features of food allergens is a high degree of resistance to digestion by pepsin in the stomach.^[^ ^48^ ^]^ Here, we showed that rMYL1 was able to resist the proteolytic action of pepsin during 60 minutes of in vitro simulated gastric digestion. On the other hand, rMYL3 was completely hydrolyzed by this time point and its degradation started already after 4 min of peptic digestion. The lower stability of rMYL3 to peptic digestion might be explained by the presence of a higher number of pepsin cleavage sites than in rMYL1. Based on these findings, it can be concluded that MYL1 can reach the intestine as an intact molecule, but not MYL3. This can be another reason for the higher number of individuals sensitized to MYL1 than to MYL3.

Although rMYL1 resisted gastric digestion, it was rapidly hydrolyzed once it was exposed to the pancreatic enzymes trypsin and chymotrypsin. However, food allergens are not ingested alone. Instead, allergenic proteins in food are generally embedded within a complex structure, the so‐called food matrix. This food matrix, its chemical composition, and the interactions of the allergens with the rest of the food components can, in fact, alter the resistance of the allergen to digestion.^[^ ^49^ ^]^ Indeed, we showed that the food matrix protected MYL1 from the action of the pancreatic enzymes when the in vitro digestion was performed with a whole piece of cooked beef instead of individual proteins. Under these conditions, a protein band of approximately 22 kDa, was recognized by the anti‐rMYL1 specific antibody and also by a pool of patients’ sera in the aliquots collected during the simulated gastric digestion. Furthermore, in the aliquots corresponding to the duodenal phase of the in vitro digestion, a protein band of about 20 kDa was also bound by a rMYL1‐specific antiserum and patients’ IgEs even 2 and 5 min after addition of the pancreatic enzymes. The IgE binding to the 22 and 20 kDa bands was inhibited in both cases by preincubation of the pool of patients’ sera with rMYL1, confirming that the protein recognized by patients’ IgE was indeed MYL1. It is very likely that other components of the meat‐food matrix, impaired the digestibility of MYL, thereby increasing the stability of this protein to digestion.^[^ ^50^ ^]^

Once an allergen is able to reach the intestine intact, if the protein or at least its peptides carrying IgE epitopes (which range between 6 and 15 amino acids^[^ ^48^ ^]^) are capable of crossing the intestinal epithelium, this allergen could cause allergic sensitization. Furthermore, in allergic sensitized individuals, the allergen reaching the blood stream could elicit systemic allergic reactions. It is known that some of the beef proteins like serum albumin, and oligopeptides with certain hydrophobic characteristics can be transported intact through the intestinal cells.^[^ ^51^, ^52^, ^53^ ^]^ MYL1 has indeed certain hydrophobicity, since 45.31% of its amino acids are hydrophobic, whereas 16.15% are acidic, 13.02% basic, and 25.52% neutral. We assessed whether rMYL1 could be transported intact across the intestinal epithelium by applying undigested and digested rMYL1 to a Caco‐2 cell monolayer grown on permeable inserts. The presence of rMYL1 in the basolateral medium of the cells after overnight incubation confirmed that rMYL1 was transported through the monolayer of Caco‐2 cells. Interestingly, a higher amount of protein was detected in the basolateral medium of the cell monolayer after application of rMYL1 that had been subjected to 60 min of simulated gastric digestion. It can be excluded that pepsin, that had been added to simulate gastric digestion, damaged the integrity of the Caco‐2 cell monolayer, because the solution applied to the Caco‐2 cells, had a pH of 7.4, where pepsin is inactive. Furthermore, TEER values, and therefore the integrity of the cell monolayer, did not decrease after incubation with digested rMYL1. Therefore, we hypothesize that partial peptic digestion of the protein increases its hydrophobicity, facilitating its transport across the intestinal cell monolayer.

In summary, we identified bovine myosin light chain 1 and 3 as new beef allergens. Recombinant MYL1 and MYL3 were produced in *E. coli* as folded, IgE reactive proteins. Both molecules showed high thermal stability. Whereas rMYL1 resisted 60 min of peptic digestion, rMYL3 was faster hydrolyzed by pepsin, suggesting that patients reacting with MYL3 might be primarily sensitized to MYL1. The increased stability of MYL1 caused by incorporation of myosin light chains into a food matrix and the ability of the protein to be transported across the intestinal epithelium would also explain the sensitizing capacity of MYL1 in beef sensitized individuals.

## 4 Experimental Section

### Human Sera

Sera were obtained from 31 patients with IgE antibodies to bovine meat at the Hospital Universitario La Paz (Madrid, Spain) after informed consent of the patients and with the approval by the Ethics Committee of La Paz University Hospital (Madrid, Spain) (EK565/2007). Patients with IgE antibodies to beef extracts in the ImmunoCap assay (Thermo Fisher Scientific, Uppsala, Sweden) were included in the study. The demographic, clinical, and serological characteristics of the patients were detailed in Table 1. Interestingly, besides beef, some patients also had IgE antibodies to pork and/or lamb meat.

### Meat Extracts

Five grams of raw and cooked (20 min at 95 °C) bovine or porcine meat were homogenized by freezing the samples in liquid nitrogen and subsequently grinding them to a fine powder using a mortar and a pestle. Proteins were extracted from the tissue powder with phosphate buffered saline (PBS) pH 7.5 at 4 °C with constant shaking overnight. The samples were then centrifuged at 4500 × *g* for 30 min at 4 °C and the supernatants were collected, filtered through sterile filters (0.45 µm), and stored at a −20 °C. Total protein concentrations were determined using a protein assay kit (Bio‐Rad Laboratories, Hercules, CA, USA) based on the Bradford method.^[^ ^54^ ^]^

### SDS‐PAGE and Immunoblot Analysis

Proteins from the different extracts (≈20 µg cm^−1^ gel) were separated by 12% SDS–polyacrylamide gel electrophoresis (PAGE)^[^ ^55^ ^]^ or 16.5% Tris‐tricine gels^[^ ^56^ ^]^ and either subsequently stained with Coomassie brilliant blue or blotted onto nitrocellulose membranes (GE healthcare, Little Chalfont, UK).^[^ ^57^ ^]^ Membranes were blocked with 0.5% Tween‐20 in PBS (PBST) containing 0.1% human serum albumin (HSA) and incubated overnight with the sera from patients sensitized to beef. As a negative control, the serum from an individual not sensitized to meat was used. The serum of each patient was diluted 1:10 in PBST containing 0.1% HSA. After washing with PBST, the blots were incubated for 1 h at room temperature (RT) with an HRP‐labeled mouse anti‐human IgE antibody purchased from Southern Biotech (Southern Biotech, Birmingham, AL, USA), diluted 1:5000 in PBST with 0.1% HSA. Blots were washed with PBST, and detection of IgE reactive protein bands was done using the SuperSignal West Pico Chemiluminescent Substrate (Thermo Fisher Scientific) on the FluorChem E Protein simple device (Biozym Scientific GmbH, Hessisch‐Oldendorf, Germany). When performing inhibition immunoblots, sera were preincubated overnight with 40 µg mL^−1^ of recombinant myosin light chain proteins, or for control purposes, with buffer only, before incubation with the membranes. Otherwise, experiments were performed as described for the immunoblots.

For specific detection of myosin light chain, the blots were incubated with a rabbit antiserum generated against recombinant myosin light chain (rMYL1) (dilution 1:10 000 in PBST with 0.1% HSA) and with HRP‐labeled goat anti‐rabbit secondary antibody (Vector laboratories, Burlingame, CA, USA) diluted 1:10 000. Detection of bound IgG was carried out as described above for the IgE immunoblot. The rabbit antiserum was raised against rMYL1 by immunizing a New Zealand White rabbit with the purified rMYL1 protein (by injecting three times 200 µg each of rMLY1, using once Freund's complete and twice Freund's incomplete adjuvant) by Charles River Laboratories (Kissleg, Germany).

### Liquid Chromatography Mass Spectrometry (Nano LC‐ESI MS/MS) for Identification of Beef Allergens

IgE‐reactive protein bands were excised from Coomassie Blue stained SDS‐PAGE gels. After washing and destaining, proteins fixed in the gel were reduced with dithiothreitol and alkylated with iodoacetamide.^[^ ^58^ ^]^ In‐gel digestion was performed either using the In‐Gel Tryptic Digestion kit (Thermo Fisher Scientific), or with trypsin (Trypsin Gold, Mass Spectrometry Grade, Promega, Madison, WI, USA) using a final trypsin concentration of 20 ng µL^−1^ in 50 mM aqueous ammonium bicarbonate and 5 mM CaCl_2_ for 8 h at 37 °C.^[^ ^59^ ^]^ Afterwards, peptides were extracted with 5% trifluoroacetic acid (TFA) in 50% aqueous acetonitrile supported by ultrasonication. Extracted peptides were dried down in a vacuum concentrator (Eppendorf, Hamburg, Germany) and then resuspended in 0.1% TFA.

The extracted peptides were then injected into an Ultimate 3000 RSLC system (Dionex), using a 25 cm Acclaim PepMap C18 column (Thermo Fisher Scientific) for separation, which was coupled to a TripleTOF 5600 instrument (Sciex).^[^ ^60^ ^]^ A UniProt database with the species *Bos taurus* (txid 9913) in combination with the cRAP contaminant‐database was used for identification of the proteins.

### cDNA Cloning and Expression of Recombinant Bovine and Porcine Myosin Light Chain 1 and 3

The sequences coding for bovine myosin light chain 1 (MYL1) and myosin light chain 3 (MYL3) were amplified by PCR from cow's skeletal muscle cDNA purchased from Zyagen (San Diego, CA, USA) using specific primers based on the sequences published of MYL1 (UniProt accession number A0JNJ5) and MYL3 (UniProt accession number P85100). For MYL1 the following primers were used: primer MYL1_FW (5’‐GGT GGT *CAT ATG* GCA CCA AAG AAG GAC GTA AAG AAA CC–3’) and primer MYL1_RV (5’ ‐ GGT GGT *CTC GAG* TCA GTG ATG ATG ATG ATG ATG GTT AGA CAT GAT GTG CTT GAC AAA AG – 3’). Primer MYL1_FW introduced a *Nde*I recognition site (italics) at the 5’‐end, whereas primer MYL1_RV added the coding sequence of a hexahistidine (His6) purification tag (underlined) and an *Xho*l recognition site (in italics) at the 3’‐end of the cDNA clone. For MYL3 the primers used were MYL3_FW (5’ ‐ GGT GGT *CAT ATG* GCT CCC AAA AAG CCA GAT CCC AAG – 3’), with a recognition site for the endonuclease *Nde*I (in italics) at the 5’‐end of the cDNA clone, and MYL3_RV (5’ ‐ GGT GGT *CTC GAG* TCA GTG ATG ATG ATG ATG ATG GCC GGC CAT GAT GTG CTT GAC AAA CG – 3’) with a recognition site for the endonuclease *Xho*I (italics) and a His6 tag (underlined) at the 3’‐end of the cDNA clone.

The obtained cDNAs coding for the bovine myosin light chain isoforms MYL1 and MYL3, were subcloned into the *Nde*I/*Xho*I sites of the expression vector pET‐17b (Novagen, Darmstadt, Germany) and the MYL1 and MYL3 constructs were verified by sequencing (Microsynth, Balgach, Switzerland). The constructs were then transformed into the *E. coli* strain BL‐21 (DE3), to express the recombinant proteins (rMYL1 and rMYL3). Protein synthesis was induced in liquid cultures at an OD_600_ of 0.5 with the addition of isopropyl‐β‐D‐thiogalactoside (0.5 mM) and rMYL1 and rMYL3 were expressed as soluble, His6‐tagged proteins. The proteins were later purified by immobilized metal ion affinity chromatography (IMAC) using a Protino Ni‐NTA agarose column (Macherey‐Nagel). The purity of the proteins was evaluated by Coomassie brilliant blue staining of 15% SDS polyacrylamide gels and the protein concentration was determined using a Pierce Micro BCA kit (Thermo Fisher Scientific) using BSA as a standard.

### Circular Dichroism (CD) Analysis

CD spectra of purified rMYL1 and rMYL3 (both 0.2 µg µL^−1^) were recorded at 20 °C on a Chirascan Plus Spectrometer (Applied Photophysics Leatherhead, UK) in 10 mM sodium phosphate buffer (pH 7.0) using a quartz cuvette (Hellma Analytics, Müllheim, Germany) with a path length of 1 mm. CD spectra were recorded from 190 to 280 nm with a resolution of 0.5 nm and results were the average of three scans. The final spectra were corrected by subtracting the corresponding buffer baseline spectrum obtained under identical conditions and normalized to the number of peptide bonds by using the extinction coefficient of the measured protein at 205 nm (ε205). Results were expressed as mean residual ellipticity *θ* (deg cm^2^ dmol^−1^) at a given wavelength. Thermal denaturation and refolding experiments were performed by gradually increasing the temperature from 20 to 90 °C with a heat rate of 1 °C per minute and cooling back to 20 °C. Every 1 °C, single continuous wavelength spectra were recorded, and before heating and after re‐cooling three continuous spectra were recorded.

### Enzyme Linked Immunosorbent Assay (ELISA)

Polystyrene microtiter plates (Maxisorp Nunc, Roskilde, Denmark) were coated with rMYL1 and rMYL3 with a concentration of 4 µg mL^−1^ in bicarbonate buffer (pH 9.6) (100 µL per well) and incubated overnight at 4 °C. After washing with Tris buffered saline, 0.5% v/v Tween 20 (TBST), plates were blocked with TBST containing 0.1% HSA for 2.5 h at 37 °C. Then 100 µL of each patient´s serum, diluted 1:5 in TBST, were added to the wells of the ELISA plates and the plates were incubated overnight at 4 °C. Control wells were incubated with the serum of an individual who was not sensitized to meat or with TBST only. After washing with TBST, 100 µL per well of HPR‐labeled mouse anti‐human IgE antibody (diluted 1:2000 in 0.1% TBST) were applied and then the plates were incubated for 1 h at 37 °C followed by 1 h at 4 °C. After washing with TBST, a solution of 1.7 mM 2,2'‐azino‐bis (3‐ethylbenzothiazoline‐6‐sulphonic acid) (ABTS), a horseradish peroxidase substrate, 60 mM citric acid, 77 mM Na_2_HPO_4_∙2H_2_O, and 3 mM H_2_O_2_ were applied to each well and the absorbance was measured at 405 nm in an ELISA reader (Multiskan FC, Thermo Fisher Scientific). All experiments were conducted in duplicates and the results were expressed as mean values. Values were considered positive when exceeding the value of the negative controls plus threefold the standard deviation.

### In Vitro Digestion of Recombinant Myosin Light Chain 1 and 3

The simulated gastrointestinal digestion of the bovine rMYL1 and rMYL3 proteins was carried out following the method described by Moreno et al.^30^ For this, 3.5 mg of the lyophilized proteins were dissolved in simulated salivary fluid (5 mM potassium phosphate, 4 mM CaCl_2_, 0.04% NaCl, pH 6.5) at 37 °C. The pH was adjusted to 3 with HCl and after 2 min, an aliquot representing the oral phase was taken. All the following steps were carried out incubating the mixture at 37 °C while shaking (150 rpm). Gastric digestion started after the addition of pepsin (Sigma–Aldrich, Vienna, Austria) at a physiological enzyme to substrate ratio of 182 U mg^−1^ of protein. Aliquots were taken after 1, 2, 4, 6, 8, 10, 15, 20, and 60 min of incubation after the addition of pepsin. In the collected aliquots pepsin was immediately inactivated by increasing the pH to 7.5 by addition of 1 M NaHCO_3_. The aliquots were then stored at −20 °C. Bis‐Tris (0.25 M final concentration, pH 6.5), and CaCl_2_ (7.6 mM final concentration) were added to the digestion mixture and subsequently the pH was adjusted to 7 with 1 M NaHCO_3_. For the simulated duodenal digestion, pancreatic bovine trypsin (EC 232‐650‐8, type I 10 100 BAEE U mg^−1^ protein, Sigma–Aldrich) and pancreatic bovine α‐chymotrypsin (EC 232‐671‐2; type I‐S; 55 U mg^−1^ protein, Sigma–Aldrich) were added to the sample at enzyme to substrate ratios of 34.5 and 0.44 U mg^−1^ protein, respectively.^[^ ^31^ ^]^ Aliquots were taken 5, 15, 30, and 60 min after the addition of the pancreatic enzymes and digestion was stopped by addition of Pefabloc SC (Sigma–Aldrich) at a final concentration of 5 mM to each aliquot.

### In Vitro Digestion of Cooked Beef

Five grams of cooked beef were digested according to the COST INFOGEST network harmonized protocol^[^ ^32^ ^]^ with minor modifications. Differently to the COST INFOGEST protocol, the gastric and the duodenal digestions were both carried out for 60 min. All enzymes (amylase, pepsin, and pancreatin) were purchased from Sigma Aldrich.

Mastication was simulated by mincing the meat and successively mixing it with 3.5 mL of simulated salivary fluid (SSF) electrolyte stock solution, 25 µL of CaCl_2_ (0.3 M final concentration), 0.5 mL of amylase solution (1500 U mL^−1^ final concentration) and H_2_O to a final volume of 10 mL. The mixture was then incubated for 2 min at 37 °C while shaking and an aliquot of 0.5 mL representing the oral phase, was taken (O). The simulated oral bolus was mixed with 5.2 mL of simulated gastric fluid (SGF) electrolyte stock solution and 4.9 µL of CaCl_2_ (0.3 M final concentration). The pH of the mixture was lowered to 2.6 and water was added to a final volume of 20 mL. Then 0.5 mL of pepsin dissolved in SGF (2000 U mL^−1^ final concentration) were added and aliquots were taken after 5, 10, 15, 30 and 60 min (G5, G10, G15, G30, G60). The pH of each aliquot was immediately increased with Na_2_HCO_3_ to a pH of 7 or 7.5 to inactivate the pepsin and each aliquot was immediately frozen in liquid nitrogen. After 60 min of incubation, 10.4 mL of simulated intestinal fluid (SIF) and 31 µL of CaCl_2_ (0.3 M in the final volume) were added to the rest of the sample and the pH was raised to 6.5–7.0 with Na_2_HCO_3_, to stop the pepsin activity. Bile salts in a final concentration of 10 mM, 2 mL of 8xUSP (U.S. Pharmacopeia) pancreatin dissolved in SIF (amount providing a trypsin activity of 100 TAME U mL^−1 [^ ^32^ ^]^) and H_2_O were added to reach a final volume of 30 mL. 0.5 mL aliquots of this duodenal phase were taken 2, 5, 15, 20 and 60 min (D5, D10, D15, D20, D60) after addition of pancreatin. The enzymatic activity was stopped in each aliquot by adding Pefabloc (5 mM final concentration) and the aliquots were subsequently frozen in liquid nitrogen.

The stock solutions of the different fluids consisted of 15.1 mM KCl, 3.7 mM KH_2_PO_4_, 13.6 mM NaHCO_3_, 0.15 mM MgCl_2_(H_2_O)_6_, 0.06 mM (NH_4_)_2_CO_3_ in case of SSF; 6.9 mM KCl, 0.9 mM KH_2_PO_4_, 25 mM NaHCO_3_, 47.2 mM NaCl, 0.1 mM MgCl_2_(H_2_O)_6_, 0.5 mM (NH_4_)_2_CO_3_ in case of SGF and 6.8 mM KCl, 0.8 mM KH_2_PO_4_, 85 mM NaHCO_3_, 38.4 mM NaCl, 0.33 mM MgCl_2_(H_2_O)_6_ in case of SIF.

For evaluation of the digestion products, the thawed aliquots were first centrifuged for 5 min at 16 000 × *g* to remove particulate matter, then the supernatants were analyzed on Coomassie stained 15% SDS‐PAGEs, on 16.5% Tris‐tricine gels, in immunoblots or in inhibition immunoblots. For detection of myosin light chain, immunoblots were performed using the anti‐rMYL1 rabbit antiserum and for detection of IgE reactive proteins, IgE immunoblots were carried out with a pool of 5 sera from patients sensitized to bovine meat (patients 23, 24, 25, 26, and 27), as described above for the immunoblot analysis. For preparation of the serum pool equal volumes of each serum were mixed and the serum pool was then diluted 1:10 in PBST containing 0.1% HSA. To prove the specificity of the IgE binding, also inhibition immunoblots were done. For this, the serum pool was preincubated overnight with 40 µg mL^−1^ of rMYL1 before incubation with the membranes. Otherwise, experiments were performed as described for the immunoblots. To assess the degree of inhibition, the intensities of the signals were analyzed using ImageJ software (National Institutes of Health, Bethesda, MD, USA).

### Intestinal Transport Experiments Using Caco‐2 Cells

Human epithelial colorectal adenocarcinoma cells, Caco‐2 (300 137, CLS GmbH, Eppelheim, Germany) were cultured in T‐175 flasks in high glucose (25 mM) Dulbecco's Modified Eagle's Medium (DMEM) (Sigma) supplemented with 10% fetal bovine serum (FBS, Gibco), 2% L‐glutamine and 1% Penicillin‐Streptomycin in a humidified atmosphere with 5% CO_2_ at 37 °C as described before.^[^ ^61^ ^]^ Once the flasks reached 80–90% confluence, the cells were split and seeded onto PET membranes of ThinCert tissue culture inserts (0.4 µm pore size, 24 mm diameter, Greiner Bio‐One, Kremsmünster, Austria) at a density of 4.3 × 10^5^ cells/insert. Medium was changed every second day and cells were used for experiments after 21 days, when a tight monolayer of fully differentiated cells was formed. The formation of tight monolayers was assessed by measuring the transepithelial electrical resistance (TEER) before and after the incubation with the extracts using the EVOM resistance meter (World Precision Instruments, Sarasota, FL, USA).

For the transport experiments, undigested and digested rMYL1 was diluted to a concentration of 150 µg mL^−1^ in DMEM containing 0.1% FBS. Then, 1.5 mL of the rMYL1 dilutions were added to the apical compartment of the cell culture inserts and Caco‐2 cells were incubated overnight at 37 °C. On the next day the different media in the basolateral chamber were collected and the presence of rMYL1 was assessed by immunoblots with the anti‐rMYL1 rabbit antiserum.

## Conflict of Interest

The authors declare no conflicts of interest.

## Supporting information

Supporting Information

Supporting Information

## Data Availability

The data that support the findings of this study are available from the corresponding author upon reasonable request.
