# Supplementary material for: Bos d 13, A Novel Heat‐Stable Beef Allergen
Source: Mol Nutr Food Res. 2023 Jun 30;67(16):2200601. doi: 10.1002/mnfr.202200601 (PMC10909433; doi:10.1002/mnfr.202200601)
Supplement: Supplementary file 1 — Supporting Information [file MNFR-67-2200601-s001.pdf]

Supplementary Figure S1

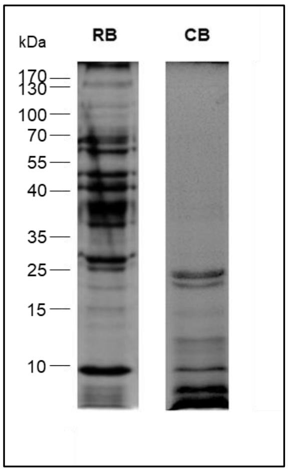

Figure S1. Analysis of the effect of cooking on bovine meat proteins. Extracts from raw (RB) and cooked (CB) beef separated by SDS-PAGE and stained with Coomassie brilliant blue. Molecular weight markers are indicated in the left margin.

Supplementary Figure S2

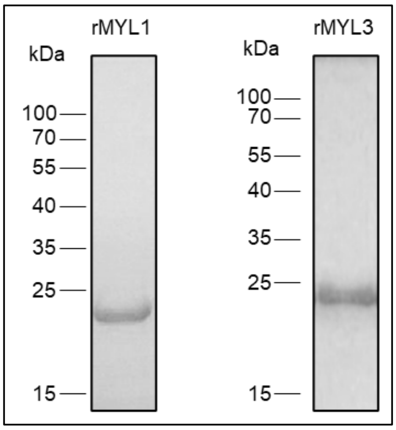

Figure S2. Analysis of the purity of recombinant myosin light chain 1 (rMYL1) and 3 (rMYL3). Hexahistidine tagged recombinant proteins purified by immobilized metal affinity chromatography were loaded on SDS-PAGE gels and stained with Coomassie Brilliant blue. Molecular weight markers are indicated in the left margin.

Supplementary Figure S3

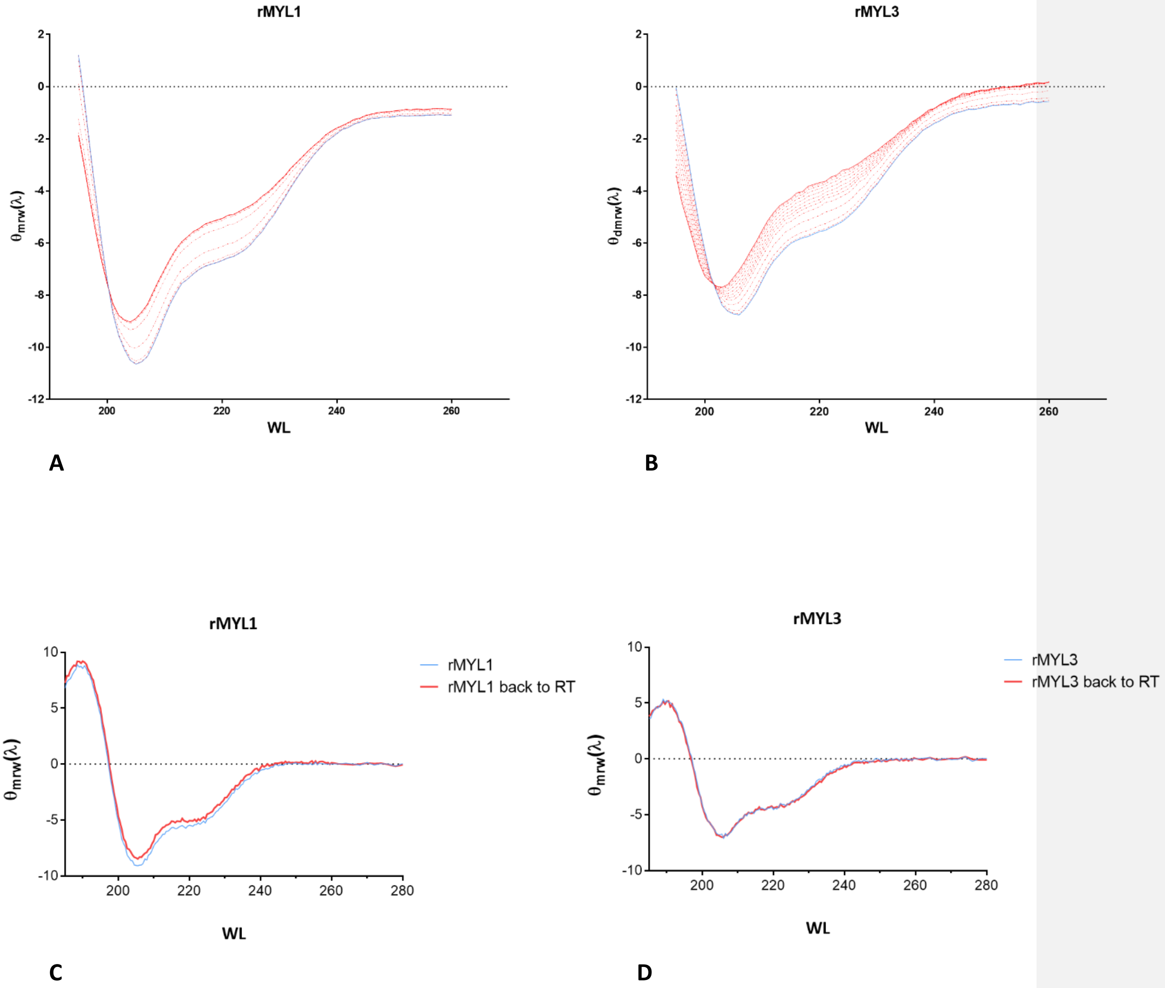

Figure S3. Effect of heat treatment on rMYL1 and rMYL3. The effect of heat treatment on the structure of rMYL1 (A, C) and rMYL3 (B, D) was determined by far-UV CD analysis. The spectra were recorded in a temperature scan from 20°C (blue line) to 90°C (strong red line) in a wavelength (WL) range from 180 to 280 nm (A, B). Panels in C and D show spectra measured before heating (blue) and after cooling back to room temperature (RT) (red). The spectra are displayed as mean residue ellipticity (deg*cm^2*dmol^{-1}, y-axis) at given wavelengths (in nm, x-axis). The spectra shown in panels C and D were corrected by shifting the spectra to the zero line.

Comment [SI1]: Please enlarge the text in panels A and B a bit!
Exchange in the legend of the y-axes always "corr" by "mrw". In A and B write WL to the x-axis!

Supplementary Figure S4

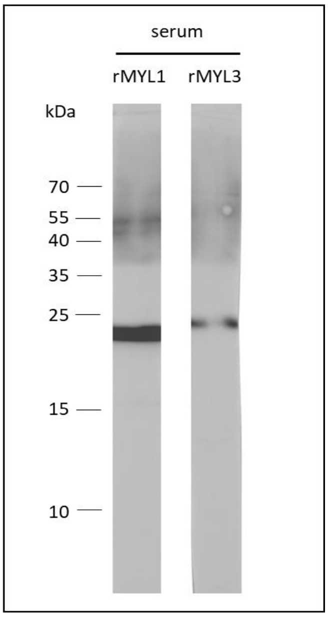

Figure S4. Analysis of the IgE reactivity of rMYL1 and rMYL3 by IgE-immunoblot. rMYL1 and rMYL3 were separated in by SDS-PAGE, blotted onto nitrocellulose membranes and exposed to the serum from a new myosin light chain sensitized patient. This serum was obtained from the company AbBaltis (FDA approved; Sittingbourne, United Kingdom). Bound IgE antibodies were detected with anti-human IgE antibodies. Molecular weights (kDa) are indicated in the left margin.
