# Supplementary material for: Bos d 13, A Novel Heat‐Stable Beef Allergen
Source: Mol Nutr Food Res. 2023 Jun 30;67(16):2200601. doi: 10.1002/mnfr.202200601 (PMC10909433; doi:10.1002/mnfr.202200601)
Supplement: Supplementary file 2 — Supporting Information [file MNFR-67-2200601-s002.pdf]

Table S1. Peptides of myosin light chain 1 and myosin light chain 3 identified by mass spectrometry after tryptic digestion of protein bands B and C.

Band B

Accessions	Names	Start Position*	Sequence
sp|P85100|MYL3_BOVIN	**Myosin light chain 3**	19	**AAAAPAPAPAPPPAPEPSK**
sp|P85100|MYL3_BOVIN	**Myosin light chain 3**	19	**AAAAPAPAPAPPPAPEPSKEPEFDPSK**
sp|P85100|MYL3_BOVIN	**Myosin light chain 3**	19	**AAAAPAPAPAPPPAPEPSKEPEFDPSKIK**
sp|P85100|MYL3_BOVIN	**Myosin light chain 3**	38	**EPEFDPSK**
sp|P85100|MYL3_BOVIN	**Myosin light chain 3**	46	**IKIEFTPEQIEEFK**
sp|P85100|MYL3_BOVIN	**Myosin light chain 3**	46	**IKIEFTPEQIEEFKEAFTLFDR**
sp|P85100|MYL3_BOVIN	**Myosin light chain 3**	48	**IEFTPEQIEEFK**
sp|P85100|MYL3_BOVIN	**Myosin light chain 3**	48	**IEFTPEQIEEFKEAFTLFDR**
sp|P85100|MYL3_BOVIN	**Myosin light chain 3**	60	**EAFTLFDR**
sp|P85100|MYL3_BOVIN	**Myosin light chain 3**	75	**ITYGQCGDVLR**
sp|P85100|MYL3_BOVIN	**Myosin light chain 3**	86	**ALGQNPTQAEVLR**
sp|P85100|MYL3_BOVIN	**Myosin light chain 3**	99	**VLGKPKQEELNSK**
sp|P85100|MYL3_BOVIN	**Myosin light chain 3**	112	**MMDFDTFLPMLQHISK**
sp|P85100|MYL3_BOVIN	**Myosin light chain 3**	128	**NKDTGTYEDFVEGLR**
sp|P85100|MYL3_BOVIN	**Myosin light chain 3**	130	**DTGTYEDFVEGLR**
sp|P85100|MYL3_BOVIN; sp|A0JNJ5|MYL1_BOVIN	**Myosin light chain 3; myosin light chain 1**	143	**VFDKEGNGTVMGAELR**
sp|P85100|MYL3_BOVIN; sp|A0JNJ5|MYL1_BOVIN	**Myosin light chain 3; myosin light chain 1**	147	**EGNGTVMGAELR**
sp|P85100|MYL3_BOVIN; sp|A0JNJ5|MYL1_BOVIN	**Myosin light chain 3; myosin light chain 1**	159	**HVLATLGEK**
sp|P85100|MYL3_BOVIN	**Myosin light chain 3**	159	**HVLATLGEKLTEDEVEK**
sp|P85100|MYL3_BOVIN	**Myosin light chain 3**	168	**LTEDEVEKLMAGQEDSNGCINYEAFVK**
sp|P85100|MYL3_BOVIN; sp|A0JNJ5|MYL1_BOVIN	**Myosin light chain 3; myosin light chain 1**	176	**LMAGQEDSNGCINYEAFVK**

* Indicates the amino acid start position in myosin light chain 3

Band C

Accessions	Names	Start Position*	Sequence
sp|A0JNJ5|MYL1_BOVIN	**Myosin light chain 1**	9	**KPAAAAAPAPAPAPAPAPAPAPPK**
sp|A0JNJ5|MYL1_BOVIN	**Myosin light chain 1**	9	**KPAAAAAPAPAPAPAPAPAPAPPKEEK**
sp|A0JNJ5|MYL1_BOVIN	**Myosin light chain 1**	36	**IDLSAIKIEFSK**
sp|A0JNJ5|MYL1_BOVIN	**Myosin light chain 1**	48	**QQQDEFKEAFLLFDR**
sp|A0JNJ5|MYL1_BOVIN	**Myosin light chain 1**	55	**EAFLLFDR**
sp|A0JNJ5|MYL1_BOVIN	**Myosin light chain 1**	68	**ITLSQVGDVLR**
sp|A0JNJ5|MYL1_BOVIN	**Myosin light chain 1**	68	**ITLSQVGDVLR**
sp|A0JNJ5|MYL1_BOVIN	**Myosin light chain 1**	79	**ALGTNPTNAEVK**
sp|A0JNJ5|MYL1_BOVIN	**Myosin light chain 1**	79	**ALGTNPTNAEVKK**
sp|A0JNJ5|MYL1_BOVIN	**Myosin light chain 1**	91	**KVLGNPSNEEMNAK**
sp|A0JNJ5|MYL1_BOVIN	**Myosin light chain 1**	92	**VLGNPSNEEMNAK**
sp|A0JNJ5|MYL1_BOVIN	**Myosin light chain 1**	123	**DQGTYEDFVEGLR**
sp|A0JNJ5|MYL1_BOVIN; sp|P85100|MYL3_BOVIN	**Myosin light chain 1; myosin light chain 3**	136	**VFDKEGNGTVMGAELR**
sp|A0JNJ5|MYL1_BOVIN; sp|P85100|MYL3_BOVIN	**Myosin light chain 1; myosin light chain 3**	152	**HVLATLGEK**
sp|P85100|MYL3_BOVIN; sp|A0JNJ5|MYL1_BOVIN	**Myosin light chain 1; myosin light chain 3**	143	**VFDKEGNGTVMGAELR**
sp|P85100|MYL3_BOVIN; sp|A0JNJ5|MYL1_BOVIN	**Myosin light chain 1; myosin light chain 3**	159	**HVLATLGEK**
sp|A0JNJ5|MYL1_BOVIN	**Myosin light chain 1**	9	**KPAAAAAPAPAPAPAPAPAPAPPK**
sp|A0JNJ5|MYL1_BOVIN	**Myosin light chain 1**	9	**KPAAAAAPAPAPAPAPAPAPAPPKEEK**
sp|A0JNJ5|MYL1_BOVIN	**Myosin light chain 1**	48	**QQQDEFKEAFLLFDR**
sp|A0JNJ5|MYL1_BOVIN	**Myosin light chain 1**	55	**EAFLLFDR**
sp|A0JNJ5|MYL1_BOVIN	**Myosin light chain 1**	68	**ITLSQVGDVLR**
sp|A0JNJ5|MYL1_BOVIN	**Myosin light chain 1**	79	**ALGTNPTNAEVK**
sp|A0JNJ5|MYL1_BOVIN	**Myosin light chain 1**	79	**ALGTNPTNAEVKK**
sp|A0JNJ5|MYL1_BOVIN	**Myosin light chain 1**	91	**KVLGNPSNEEMNAK**
sp|A0JNJ5|MYL1_BOVIN	**Myosin light chain 1**	92	**VLGNPSNEEMNAK**
sp|A0JNJ5|MYL1_BOVIN	**Myosin light chain 1**	123	**DQGTYEDFVEGLR**

* Indicates the amino acid start position in myosin light chain 1
